# Splinting after Carpal Tunnel Release: Does it really Matter?

**DOI:** 10.5704/MOJ.1507.011

**Published:** 2015-07

**Authors:** A Shalimar, MH Nor-Hazla, A Arifaizad, S Jamari

**Affiliations:** Department of Orthopaedics and Traumatology, Faculty of Medicine, Universiti Kebangsaan Malaysia, Cheras, Kuala Lumpur, Malaysia

**Keywords:** Carpal tunnel syndrome, carpal tunnel release, wrist splint, limited open carpal tunnel release, nerve compression

## Abstract

Splinting of the wrist after carpal tunnel release (CTR) has been practised by many surgeons especially in North America. The main reason was to prevent possible adverse events of bowstringing of flexor tendons and the median nerve, pillar pain, entrapment of the median nerve in scar tissue and wound dehiscence. Studies on the effect of splinting after standard CTR have had dismal results. The duration of splinting in standard CTR has been either too long (for 2-4 weeks) or too short (48 hours only). The aim of our study was to compare the effects of post-operative splinting for a duration of one week with no splinting.

Methods: All 30 of our patients underwent a standardized limited open CTR by a designated surgeon. Post operatively, they were randomized into a splinted (n=16) and a nonsplinted (n=14) group. The splint was kept for a week. Patients were reviewed at regular intervals of one week, two months and six months. At each follow up, these patients were clinically assessed for the following outcome measures: VAS (visual analogue score), 2PD (two-point discrimination), pinch grip, grip, Abductor Pollicis Brevis (APB)) power and completion of the Boston questionnaire. Results: All patients presented with significant improvement in the postoperative evaluation in the analyzed parameters within each group. However, there was no significant difference between the two groups for any of the outcome measurements at sequential and at final follow-up.

Conclusion: We conclude that wrist splinting in the immediate post-operative period has no advantage when compared with the unsplinted wrist after a limited open carpal tunnel release.

## Introduction

Carpal tunnel syndrome is the most common entrapment neuropathy often occurring after the age of 30 years, with women affected three to six times more than men^1,2^. Limited open carpal tunnel release is effective in treating carpal tunnel syndrome with a cure rate of 90% and less associated complications compared to traditional open carpal tunnel release. However, even with a smaller scar, post-operative pain still occurs with an incidence of up to 48%^3,4^.

Post-operative wrist splinting is from a neutral position to 15º of extension and places the carpal tunnel in its most open position. This allows maximal circulation to the median nerve, prevents compression during activities, bowstringing of flexor tendons, wound dehiscence, reduction of pillar pain and entrapment of the median nerve within the surgical incision^5-8^.

One to two weeks of splinting have been recommended following endoscopic carpal tunnel release^5,9^ and one to three weeks following open carpal tunnel release^10^. A survey of American hand surgeons found that 81% of them splinted their patients' wrists for two to four weeks following carpal tunnel surgery^7^.

Previous studies have reported splinting as ineffective, but those studies were on classical open CTR and not on limited open CTR or endoscopic CTR. Splints were applied either too long (between 2- 4 weeks^11-14^) or too short (within 48 hours^15^). Cook *et al*^12^ advocated splinting for one week as a precaution against the complications of tendon bowstringing and nerve entrapment in the scar.

The objective of our study was, firstly, to compare between splinting and no splinting after limited carpal tunnel release and its outcome on patients’ pain and functional level and, secondly, to ascertain the suitability of splinting for an in-between duration of one week.

Our null hypothesis states that there is no difference in pain score using visual analogue score in both splinting and nonsplinting groups after limited open carpal tunnel release. Also, the null hypothesis states that there is no difference in function and symptoms in both splinting and non-splinting groups after limited open carpal tunnel release.

## Materials and Methods

The study is a single centre prospective randomized controlled trial approved by our Ethical Committee conducted in the Hand and Microsurgery Unit of the Orthopaedic Department between October 2007 and June 2008.

**Inclusion and Exclusion Criteria:** All patients diagnosed with carpal tunnel syndrome and who underwent carpal tunnel release from October 2007 until June 2008 were included in this study. Carpal tunnel syndrome was diagnosed clinically based on history and physical examination. The exclusion criteria were patients with local causes of carpal tunnel e.g. tumour/ cyst in the carpal tunnel, bone piece, post fracture,; peripheral nerve condition (double crush pathology); arthritis (rheumatoid arthritis); revision surgery; pregnant patients; patients undergoing endoscopic release or standard open CTR and patients for CTR not for carpal tunnel syndrome *per se* but for other causes like decompression in compartment syndrome or replantation at mid-carpal level.

**Outcome Measures:** An independent observer blinded to the procedure collected demographic data such as age, gender, occupation, previous medical illness and hand dominance. The Boston questionnaire was employed^16^ This is a disease-specific questionnaire and has two parts. The first part assesses the severity of symptoms and the second part assesses functional status.

Digital sensibility was measured by the static two-point discrimination, and quantitative Phalen tests. The quantitative Phalen test was considered positive if the median nerve-distribution sensibility was diminished by one level in the gravity-assisted position of flexion of the wrist. Tinel sign was elicited and noted. Thenar bulk and contour was assessed with atrophy recorded as 0 (absent), 1 (mild), 2 (moderate), or 3 (severe). The strength of the Abductor Pollicis Brevis was measured and graded from M0 to M5, according to the criteria of the Medical Research Council muscle power grading. Grip strength was measured with a JAMAR dynamometer (Sammons Preston Rolyan, Chicago, Illinois) together with key pinch strength (Sammons Preston Rolyan, Chicago, Illinois); these values were recorded for the affected hand and the contralateral hand.

A preliminary nerve conduction study was not considered essential in our patients. However if it had been done, the findings were noted. An informed consent was obtained prior to inclusion in the study. The indication for surgical intervention was clearly defined either failed conservative and/or definite motor and/or sensory deficit. The surgical procedure, randomization and post-operative splinting or non-splinting protocols were explained to the patients.

To decrease surgical bias, a single designated surgeon was chosen to perform the surgery under general anaesthesia. However we were unable to blind patients post operatively. Patients were seen by the designated surgeon during the first follow up for appropriate care. Subsequently the patients were seen by a blinded researcher for assessment.

Surgical Procedure: The length of the incision was between 1.5 cm to 2.0 cm^3,4^ distal to the most prominent distal wrist crease and did not extend beyond the Kaplan line. Neurolysis and tenosynovectomy were not done. Skin was approximated using non-absorbable nylon (Ethilon®) 6/0 and dressed with fluffy gauze.

Post-operative Protocol: Patients were randomized into two groups immediately post-operative. One group was applied with a volar plaster splint (8 layers of Gypsona ®) for one week with the wrist in a 15º extended position and full finger motion allowed. The splints extend from the mid-half of the forearm till the metacarpophalangeal level allowing tendon and nerve gliding exercises. A plaster template was used to ensure the splints were uniform. The second group had a soft bulky dressing applied for a week with unrestricted active motion. Analgesics for both groups were similar. All patients were seen at one week, two months and six months after the operation.

**Statistical Analysis:** Data was analyzed using statistical package SPSS for Windows Version 13 using the student t and Chi-square test. Further analysis was assisted by a statistician. Taking the power of the study at 80%, with a confidence interval of 95%, a p value of <0.05 was considered significant.

## Results

There were 30 hands from 26 patients (four patients with bilateral release of which three were done simultaneously and one sequentially) enrolled into this study. There were five (19%) males and 21 (89%) females. Their age ranged from 32 to 65 years old (Table I). All patients were accounted for and were followed up till the last required follow up.

**Table I:** Demographic details and clinical tests comparing splinted and non-splinted (NS) groups to ensure similar demographic and clinical parameters. Note that the number in brackets are percentages

		Splint		Pearson Chi-Square	P value
		Splint	No Splint		
Age (mean)		49.8±8.7	49.3±8.5		0.88
Sex	Male	4 (67)	2 (33)	0.54	0.46
	Female	12 (50)	12 (50)		
Dominance	Right	15 (56)	12 (44)	0.54	0.46
	Left	1 (33)	2 (67)		
Operated hand	Right	10 (59)	7 (41)	0.48	0.49
	Left	6 (46)	7 (54)		
Occupation	House wife	5 (63)	3 (38)	3.98	0.41
	Labour	6 (55)	5 (45)		
	Clerk/admin	3 (38)	5 (63)		
	Teacher	0	1 (100)		
	Other	2 (100)	0		
Worker’s Comp	Yes	5 (71)	2 (29)	1.20	0.27
	No	11 (48)	12 (52)		
NCS	Done	11 (52)	10 (48)	0.03	0.87
	Not done	5 (56)	4 (44)		
Phalen test	Yes	13 (62)	8 (38)	2.07	0.15
	No	3 (33)	6 (67)		
Tinel sign	Yes	12 (57)	9 (43)	0.41	0.52
	No	4 (44)	5 (56)		
Diabetes	Yes	3 (50)	3 (50)	0.03	0.85
	No	13 (54)	11 (46)		
Smoking	Yes	1 (100)	0	0.91	0.34
	No	15 (52)	14 (48)		
Hypertension	Yes	6 (55)	5 (45)	0.01	0.92
	No	10 (53)	9 (47)		

The 30 hands enrolled in this study (of which 16 were splinted and 14 non-splinted) did not significantly differ with respect to any demographic or clinical variable (all parameters had p value > 0.05) (Table II). Nerve conduction studies were done on 10 (71%) hands in the non splinted group and 11 (69%) hands in the splinted group. The Phalen test and Tinel sign were positive in 58% and 64% in the nonsplinted group and 81% and 75% respectively for the splinted group.

**Table II:** Outcome data comparing splinted and non-splinted groups

		Preop		Immediate		1 wk		2 mth		6 mth	
		Splint	NS	Splint	NS	Splint	NS	Splint	NS	Splint	NS
VAS				4.7±2.0	5.6±2.3	2.6±1.5	2.6±2.1	1.3±1.8	1.4±1.8	0.1±0.3	0.4±1.3
2pd	radial	7.0±3.7	6.4±2.0			5.3±1.1	5.7±1.4	5.0±1.0	5.6±1.4	4.3±1.0	4.7±0.7
	ulna	5.8±1.6	6.2±1.6			5.6±2.0	6.2±1.6	4.8±0.5	5.6±1.3	4.3±1.0	5.3±2.1
Pinch		4.9±2.0	4.5±1.5					4.3±1.5	5.1±1.4	6.0±2.4	6.2±1.9
Grip		18.4±8.2	18.9±4.5					16.3±6.9	18.5±4.9	21.4±8.5	22.9±5.6
APB		3.9± 0.7	3.7±1.1			3.8±0.7	3.5±0.9	4.1±0.4	4.1±0.4	4.5±1.1	4.4±1.1
Boston score	Part I	2.6±0.7	2.5±0.5			1.6±0.4	1.9±0.5	1.2±0.2	1.4±0.3	1.0±0.0	1.1±0.3
	Part II	2.9±0.5	2.8±0.8					1.5±0.5	1.4±0.5	1.0±0.1	1.0±0.4

VAS, Visual analogue score; 2pd Radial, 2 point discrimination on the radial side of the index finger; 2pd Ulna, 2 point discrimination on the ulna side of the index finger; Grip, JAMAR grip strength; Pinch, pinch strength; APB power= Medical research muscle power grade of the Abductor pollicis brevis

(p value for comparison of each value ranges from 0.07 to 0.98)

Note that the shaded areas represent for VAS (patient has no pain yet as the operation has not been performted) and for pinch, grip, APB and Boston questionnaire (patients are unable to be examined due to early post-operative pain period)

**Table III:** Outcome data comparing splinted and non-splinted (NS) groups

	Patients with scar pain		p value	Patients with pillar pain		p value
	Splint	No splint		Splint	No splint	
1 week	14	12	0.886	0	0	
2 months	4	5	0.53	1	1	0.922
6 months	0	0		0	1	0.277

Only one person was noted to have a history of smoking in the splinted group. There were five cases of diabetes mellitus, three of hypertension and one of ischaemic heart disease in both groups. One of the patients with hypertension also suffered from chronic rheumatic heart disease.

Ninety percent of all the patients were right hand dominant. The right hand was operated on 62% in the splinted group and 50% in the non-splinted. The majority of the patients (73%) were employed with only seven amongst them applying for worker’s compensation.

Patients in the non-splinted group began unrestricted active motion on the first post-operative day. Patients in the splinted group were splinted for seven days. Clinically both groups did not differ as all the parameters had a p value > 0.05. Within the group itself, there were parameters that had significantly improved

The Boston score was divided into two parts during analysis as suggested by Levine *et al*^16^. The first part consisted mainly of symptoms and the second part was the functional assessment. Both groups reported improvement -y in the Boston score. The score for both groups improved significantly (p<0.05). The splinted group had their Boston score improving from a mean of 2.6 for part I and 2.5 for part II to 1.0 and 1.1 respectively. The non-splinted group had improved - Boston score from 2.9 and 2.8 to 1.0 and 1.1 respectively. However, there was no significant difference in any parameter (p>0.05) when comparing both groups. Although English was not the first language, all the patients were able to understand the questionnaire.

**Fig. 1:**
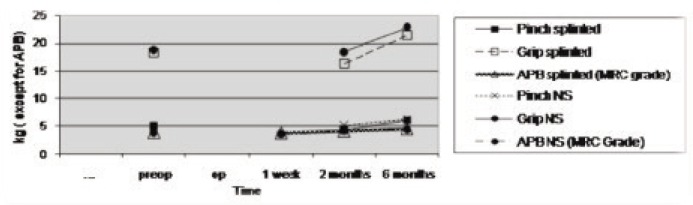
Graph of motor improvement in splinted and non-splinted (NS) groups (Pre-op: preoperative, Op: operative period).

**Fig. 2:**
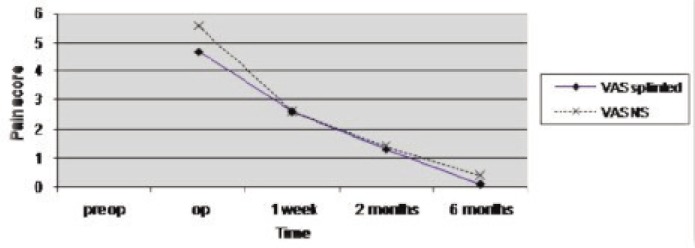
Graph of visual analogue score for pain in splinted and non-splinted (NS) groups (Pre-op: preoperative, Op: operative period).

In the non-splinted group the two-point discrimination had improved significantly (p=0.012), whereas in the splinted group the JAMAR grip strength and the pinch grip strength had improved significantly (JAMAR; p=0.037 and pinch; p=0.042) (Fig 1). These improvements were seen at one week, two months and six months post-operatively. There was no significant difference between both groups with regard to the prevalence of scar or pillar pain (p>0.05) by the VAS (Fig 2).

Even though there was no scar complication or any bowstringing noted, one patient in the non-splinted group did not have a significant improvement in her JAMAR, pinch strength and Boston score, although two-point discrimination improved with no complaint of pillar pain. Four patients with bilateral carpal tunnel release- were operated either simultaneously or sequentially. Three were randomized with a splint on one side and un-splinted on the other. The other person was splinted bilaterally. The clinical parameters and Boston score in the four patients were not significant. With regards to their preference regarding splinting and non-splinting, one preferred no splint as it was easier to freely move the hand whereas the other two preferred splinting as it made them less worried about their operated hand.

## Discussion

The present study revealed a similar demographic pattern with 89% of the patients being females. The age group range was between 32-65 years old.

A large proportion of patients who failed conservative treatment subsequently underwent carpal tunnel decompression with high success rates^2^. While patient satisfaction is usually high with surgery, potential complications do exist and includes pain and scar discomfort, wound dehiscence, bowstringing of the flexor tendons and inclusion of the median nerve within the postoperative scar^4,6,17^.

To minimise these complications, most surgeons immobilize wrists for one to four weeks following open carpal tunnel surgery.8 Conversely, some authors recommend early aggressive mobilization of wrist and fingers after surgery in order to enable - free longitudinal nerve movement in the surgical bed preventing possible adherence to neighbouring structures^18^.

A few studies reported in the literature have investigated the effects of immobilization following open carpal tunnel release^11-15^. The duration was either too long, for 2-4 weeks, or too short, for 48 hours.

Bury *et al*. compared two week of post-operative wrist splinting versus a bulky dressing after 43 open carpal tunnel releases^11^. There were no significant differences between the two groups. Evaluation included subjective parameters of patient satisfaction and objective parameters of grip and lateral pinch strength, complication rates, and digital and wrist ranges of motion^11^.

Finsen *et al.* reported no significant differences between post-operative immobilization and non-immobilization after open carpal tunnel release in 82 wrists^13^. The splint was used for four weeks and pain and scar discomfort were evaluated through a visual analogue scale and the grip and key pinch strength. Cebesoy *et al* compared two groups, the first splinted for three weeks followed by exercises and the second group with a bulky bandage and immediate postoperative exercises. They concluded that post-operative immobilisation had no detectable benefits and may cause unnecessary expenditure and discomfort^15^. Cook *et al.* did actually find significantly better results in the non-splinted group when considering subjective pain scale. There was also an earlier return in grip and key pinch strength. However the splinting was for two weeks^12^.

Bhatia *et al* splinted 45 hands for 48 hours comparing with 57 non-splinted hands. There were no significant statistical differences in the pain scores or number of analgesic tablets taken up to three days postoperatively between the two groups^14^.

Published data do not show sufficient evidence to justify routine wrist splinting following open carpal tunnel release. In the current study too, no difference was found between the two patients groups with and without a post-operative splint. The use of wrist splint following open carpal tunnel release had not been previously studied using a validated outcome questionnaire.

The limitation of the present study is the small sample population which may not detect subtle differences between the two groups. The follow up period is for six months only, whereas pillar pain has been reported to occur even after six months post operatively, albeit rarely. Another limitation of our study was that patients with diabetes mellitus were not tested for peripheral neuropathy.

Our study concludes that wrist splinting for one week postoperatively has no significant difference compared to nonsplinted wrists in patients with limited open carpal tunnel release.
